# Diagnostic Utility of N-terminal pro-Brain Natriuretic Peptide and C-reactive Protein in Diagnosing Heart Failure in Patients with Acute Hypoxemic Respiratory Failure

**DOI:** 10.7759/cureus.6835

**Published:** 2020-01-31

**Authors:** Azmat Abdullah, Ahtesham Iqbal, Wasib Ishtiaq, Syed Waqar Hussain, Aayesha Qadeer, Ghulam Rasheed

**Affiliations:** 1 Internal Medicine, Shifa International Hospital, Islamabad, PAK; 2 Critical Care, Shifa International Hospital, Islamabad, PAK; 3 Internal Medicine, Khan Research Laboratories Hospital, Islamabad, PAK

**Keywords:** n terminal pro bnp, c-reactive protein

## Abstract

Introduction

Shortness of breath is a leading cause of intensive care unit (ICU) admissions and is multifactorial. Acute hypoxemic respiratory failure due to heart failure is one of the leading causes of ICU admissions. N-terminal pro-brain natriuretic peptide (NT-proBNP) is secreted by ventricles and carries a negative predictive value for heart failure (2). NT-proBNP can also be raised in sepsis (4). Changes in NT-proBNP strongly correlated with changes in C-reactive protein (CRP) and leukocytes levels (8).

Objective

This study was conducted to explore the diagnostic utility of NT-proBNP and CRP to diagnose heart failure in patients presenting with acute hypoxemic respiratory failure.

Materials and methods

After informed consent and approval from the institutional review board (IRB), patients of acute hypoxemic respiratory failure were included in the study. History and physical examination were done by a medical resident and recorded in the patients' files. Data were transferred to a structured proforma by the researcher. All tests were conducted within three hours of presentation. The diagnosis of heart failure was made by a panel of experts, including the consultant cardiologist and consultant intensivist in charge. The chest X-ray was reported by the radiologist. The cost of the test was afforded by the institution.

Data were analyzed by SPSS version 15 (SPSS Inc., Chicago, Illinois). Analysis of variance (ANOVA), Pearson correlation and linear regression were applied to find out the relation between variables and significance.

Results

We studied 137 patients. Out of them, 72.9% were diagnosed as heart failure. Heart failure was more common in females (43.7%) as compared to males (29%). NT-proBNP was raised in 111 (81%) patients and out of them, 88 patients (79%) had heart failure. Sensitivity and specificity of NT-proBNP were found to be (95.56%) and (46.81%), respectively. Similarly, CRP was 90% sensitive and 25.53% specific for heart failure. The most common findings in chest X-rays of patients with heart failure were upper lobe diversion and enlarged cardiothoracic ratio (71%).

Conclusion

We concluded our study as NT-proBNP is a highly sensitive test to diagnose heart failure in settings of acute hypoxemic respiratory failure. CRP is also significantly raised in heart failure. Upper lobe diversion and an increased cardiothoracic ratio is a strong predictor of heart failure.

## Introduction

Shortness of breath is one of the leading reasons for ICU admissions and presentation to the emergency department (ED) worldwide. Dyspnea can be cardiac, respiratory, hematological, or metabolic in origin. The attending physician receiving such patients can usually differentiate between these causes based on history, clinical examination, and laboratory tests. However, often, it is difficult to ascertain the cause, and more sophisticated tests are required to making the diagnosis and initiate appropriate management especially in the ICU population.

Pro-brain natriuretic peptide is secreted by ventricles in response to stretch from fluid or pressure overload. This is converted to the active metabolite brain natriuretic peptide (BNP) and the inactive NT-pro-brain natriuretic peptide (NT-proBNP) by the enzyme corin. BNP and NT-proBNP are usually interchangeable, with no clinically useful difference in accuracy [1]. BNP is considered to have a very good negative predictive value for heart failure [2]. N-terminal proB-type natriuretic peptide (the inactive metabolite of proBNP) is shown to be a good marker of prognosis in patients with heart disease [3].

Various factors affect the plasma levels of these biomarkers apart from heart failure. For example, NT-proBNP is found to be markedly elevated in patients with septic shock and may not be specific to heart failure [4]. Various other conditions, such as sepsis, intracranial hemorrhage, and pulmonary embolism, also increase the plasma levels of these peptides [3,5]. In addition, NT-proBNP is excreted renally; thereby, in patients with renal insufficiency, its diagnostic utility is confounded. Because of the variety of factors affecting BNP and NT-proBNP, the role of BNP is unclear in the emergency department (ED) and intensive care unit (ICU) settings [3]. Moreover, studies done on BNP and NT-proBNP to establish their diagnostic utility and prognostic markers have not been consistent; hence, the application of BNP in the ICU setting has remained academic mostly [6].

Few studies have shown that the clinician’s awareness of these cardiac markers does not have an impact on the overall outcome of patients in the ED [7]. This has led to the decreasing use of proBNP and its metabolites in the ICU and emergency settings.

Because of the interaction of the inflammatory response with natriuretic peptides, it is suggested that BNP and N-terminal proBNP should only be used cautiously in critically ill patients. Rudiger et al. found that changes in BNP and NT-proBNP levels correlated significantly (p<0.01) with those in C-reactive protein values and with leukocyte counts [8]. In a study of 75 patients, the BNP and proBNP levels in patients with sepsis were found compatible with those in acute heart failure [9]. Similarly, Fried and colleagues concluded that these peptide levels should not be used as a sole marker to differentiate between sepsis and heart failure; however, their very high concentration in blood does suggest cardiac dysfunction in the pediatric population [10].

Our study aims to see if NT-ProBNP in combination with CXR and CRP can reliably predict cardiac dysfunction in an adult ICU setting. Because NT-proBNP is a simple test, its combination with chest x-ray and markers of inflammation can be a valuable tool in diagnosing heart failure in emergency and intensive care settings. This can guide appropriate early therapy in settings where a formal echocardiogram is not available urgently for evaluation of cardiac function.

## Materials and methods

Settings and patients

The study was approved by the institutional board review of the hospital and was performed according to the guidelines of the hospital. Informed consent was taken from all patients. This was a prospective observational study conducted in the medical ICU of Shifa International Hospital, Islamabad, from March 2018 to March 2019. A total of 139 patients were studied during this time. The aim of the study was to explore the diagnostic utility of NT-proBNP, C-reactive protein, and chest X-ray to diagnose heart failure in patients presenting with respiratory failure to the medical ICU. Patients older than 18 years of age admitted to the medical ICU with acute hypoxemic respiratory failure were included in the study. Acute hypoxemic respiratory failure was defined as a condition of having a partial pressure of oxygen (PaO2) pf < 60 mmHg on arterial blood gases and a history of shortness of breath of not more than three days. Those patients who had a history of trauma within the last five days, liver failure, and using steroids were excluded from the study.

Diagnostic procedures

The demographic data and history of all patients, including the use of diuretics, were obtained by the medical resident on duty and recorded in the patient’s electronic medical record (EMR). The following data were recorded for all patients: 1. Vital signs, including pulse rate and rhythm, systolic and diastolic blood pressure, oxygen saturation, respiratory rate, and temperature; 2. Chest X-ray; 3. Arterial blood gases; 4. C-reactive protein, complete blood count, serum creatinine, serum urea, cardiac enzymes, D-dimers, and NT-proBNP (analyzed with Roche Methodics; Roche Holding AG, Basel, Switzerland); and 5. Echocardiography. All the workup was done within three hours of presentation. All the data were then recorded on a preformed structured proforma.

Once all the above data were available, patients were seen by an expert panel, including one consultant cardiologist and one consultant intensivist. They judged the clinical data and labeled patients as heart failure. Chest X-rays were reported by consultant radiologists. Echocardiography was done by the technician on call and reported by the consultant cardiologist expert in reporting echocardiography. Cardiologists, radiologists, intensivists, and medical residents were kept blind to the study to minimize bias.

Data analysis

Data analysis was done using SPSS (version 15, SPSS Inc., Chicago, Illinois). The Pearson correlation test was applied to find out the relationship between the dependent and independent variables. Analysis of variance (ANOVA) was applied to measure the good fit model. Linear regression was applied to evaluate the impact of independent variables on dependent variables.

## Results

**Figure 1:**
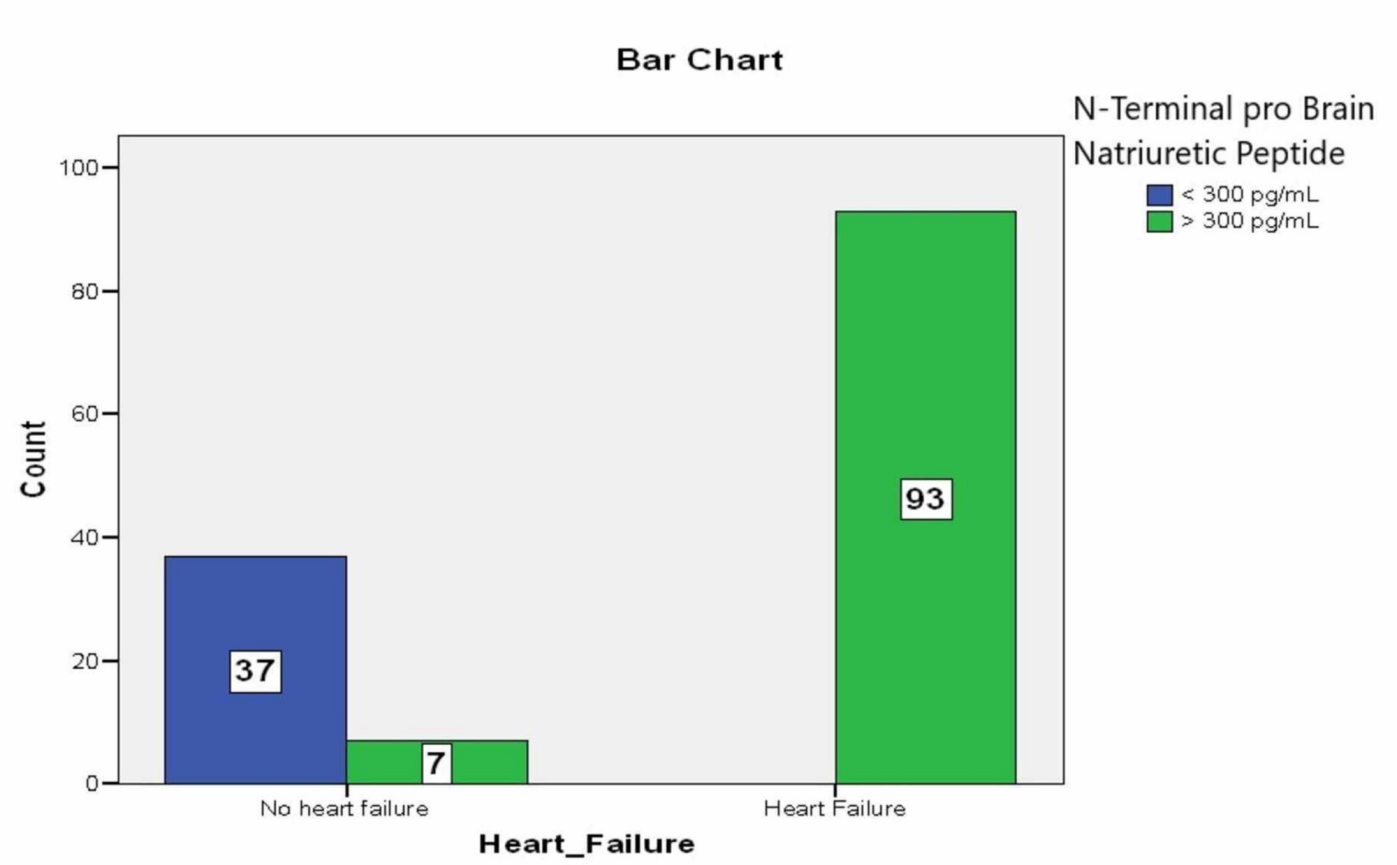
Bar chart representing a relation of N-terminal pro-brain natriuretic peptide with heart failure

**Figure 2:**
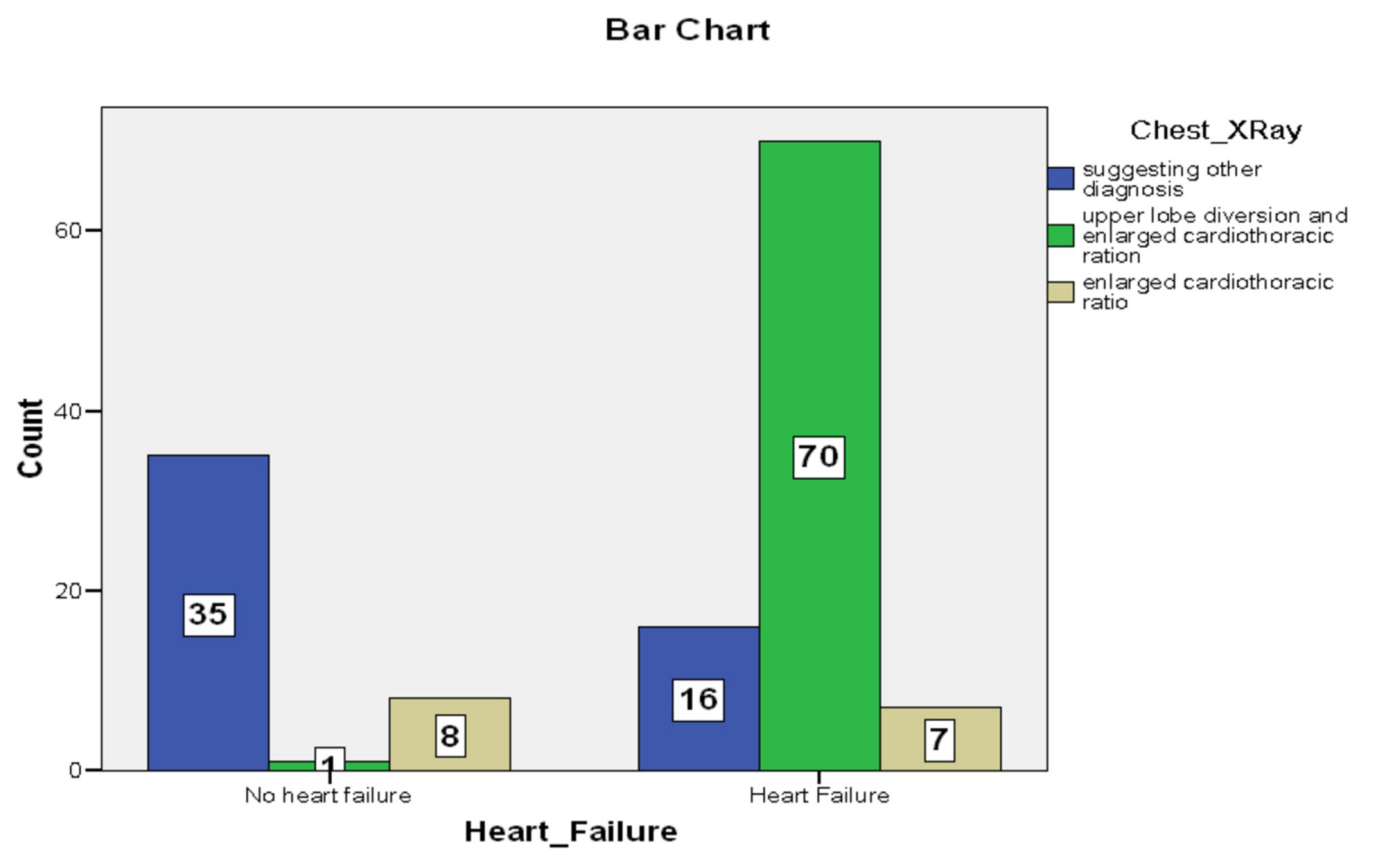
Bar chart showing X-ray findings in patients with heart failure

In our study, 137 patients (53.2% females and 46.71% males) with acute hypoxemic respiratory failure were enrolled. Among them, 72.9% were diagnosed as heart failure. Heart failure was more common in females (43.7%) as compared to males (29%). NT-proBNP was raised in 111 (81%) patients and out of them, 88 patients (79%) had heart failure (Figure 1). The sensitivity and specificity of NT-proBNP were found to be (95.56%) and (46.81%), respectively. Similarly, CRP was 90% sensitive and 25.53% specific for heart failure. The most common findings in the chest X-ray in patients of heart failure were upper lobe diversion and enlarged cardiothoracic ratio (71%) (Figure 2). We used regression analysis to evaluate the impact of independent variables on dependent variables. Our R^2^ value was 0.783, which means CRP and NT-proBNP had 78% ability to detect heart failure (p=0.001). P-value <0.05 was considered significant. See Tables 1-2 for the relationships among NT-proBNP and CRP. Table 3 shows the impact of CRP and NT-proBNP on the diagnosis of heart failure

**Table 1:** Showing the relationship between NT-ProBNP and heart failure NT-ProBNP: N-terminal pro-brain natriuretic peptide

	N Terminal-proBrain Natriuretic Peptide		Total
	< 300 pg/ml	> 300 pg/ml	
No heart failure	22 (46.8%)	25 (53.2%)	47 (100%)
Heart failure	4 (4.4%)	86 (95.6%)	90 (100%)
Total	26 (19%)	111 (81%)	137 (100%)

**Table 2:** Showing the relationship between CRP and heart failure CRP: C-reactive protein

	C-Reactive Protein		Total
	< 10 mg/L	> 10 mg/L	
No heart failure	12 (25.5%)	35 (74.5%)	47 (100%)
Heart failure	9 (10%)	81 (90%)	90 (100%)
Total	21 (15.3%)	116 (84.7%)	137 (100%)

**Table 3:** The impact of C-reactive protein and N-terminal pro-brain natriuretic peptide on the diagnosis of heart failure a. predictors: (constant), chest X-ray, C-reactive proteins, N-terminal pro-brain natriuretic peptide, diuretic therapy

Model	R	R Square	Adjusted R Square	Standard error of the estimate	Change studies				
					R Square change	F change	df1	df2	Sig. F change
1	0.885a	0.783	0.780	0.220	0.783	241.963	2	134	0.000

## Discussion

Clinical assessment, including history and examination, is mandatory for making the diagnosis of heart failure in a patient with shortness of breath. However, in many different situations, especially in ICU patients, the diagnosis often requires more objective investigations especially echocardiography (considered the standard test) along with chest X-ray, ECG, and serum BNP and NT-proBNP levels.

NT-proBNP is produced by the left ventricle, mainly in response to volume and/or pressure. When secreted, it is converted into the active BNP and inactive NT-proBNP by the enzyme named corin. While the BNP is cleared by the receptors - lungs, liver, kidneys, and vascular endothelium, NT-proBNP is cleared only by the kidneys [11]. With ventricular stretch from pressure or volume load, BNP, along with NT-proBNP, is released to increase vasodilation and sodium and water excretion through the kidneys to counter the increased fluid volume. Because of the renal clearance of these peptides, deranged renal functions may lead to increased levels of BNP and NT-proBNP [12]. Studies, however, suggest that plasma NT-proBNP levels are affected by a glomerular filtration rate (GFR) decline while BNP levels are not. They suggest BNP in chronic kidney disease patients might be more appropriate than NT-proBNP as biomarkers of cardiac dysfunction [13].

While a chest X-ray is pivotal in the emergency department in the assessment of patients with dyspnea, a meta-analysis shows that the chest X-ray is insensitive in the diagnosis of heart failure but has moderate specificity (76%-83%) [14-15]. Another study on 54 patients concluded that there was no correlation between BNP levels and findings on chest X-ray. Chest X-rays could be normal with very high BNP values [16].

NT-proBNP levels are also affected by the presence or absence of sepsis and/or inflammation. In a study done in Alexandria, the authors concluded that proBNP was elevated in patients with sepsis irrespective of the presence or absence of heart failure [17]. Studies have also shown that plasma BNP and NT-proBNP have a moderate predictive mortality value in septic patients as well [18]. Other studies, however, conclude that BNP is a reliable marker of cardiac dysfunction in patients with sepsis [19].

## Conclusions

To conclude, NT-proBNP is a highly sensitive test to diagnose heart failure in settings of acute hypoxemic respiratory failure. CRP is also significantly raised in heart failure. Upper lobe diversion and an increased cardiothoracic ratio is a strong predictor of heart failure.
